# Radiocaesium transfer and radiation exposure of frogs in Fukushima Prefecture

**DOI:** 10.1038/s41598-018-28866-0

**Published:** 2018-07-13

**Authors:** Keiko Tagami, Shigeo Uchida, Michael D. Wood, Nicholas A. Beresford

**Affiliations:** 1 National Institute of Radiological Sciences, National Institutes for Quantum and Radiological Science and Technology, Anagawa 4-9-1, Inage-ku, Chiba 263-8555 Japan; 2 School of Environment & Life Sciences, University of Salford, Manchester, M4 4WT UK; 3 Centre for Ecology & Hydrology, Lancaster Environment Center, Library Av., Bailrigg, Lancaster LA14AP UK

## Abstract

The International Commission on Radiological Protection has proposed an environmental assessment framework. This includes ionising radiation exposure assessment for different frog life-stages, but radiocaesium transfer parameters are unavailable. We collate data from the Fukushima Prefecture (contaminated by the Fukushima accident) and estimate radiocaesium concentration ratio (CR_wo-water_) values for tadpoles and adult frogs, presenting the largest available amphibian CR_wo-water_ dataset. In total, 513 adult frogs and 2540 tadpoles were analysed in 62 and 59 composite samples respectively. Results suggest that equilibrium was reached between water and amphibian radiocaesium activity concentrations *circa* one-year after the accident. Radiocaesium transfer to tadpoles was higher than to adult frogs. Dose rates were estimated for different life-stages and species in both the aquatic and terrestrial environment. Estimated dose rates to adults and tadpoles were typically similar because external exposure dominated for both organisms; frogspawn dose rates were estimated to be orders of magnitude lower than other life-stages. For the two sites assessed, which were outside of the most contaminated areas of the Fukushima Prefecture, estimated dose rates were below those anticipated to present a risk to wildlife populations; it is likely that dose rates in more contaminated areas were in excess of some effects benchmark values.

## Introduction

The developing environmental protection framework of the International Commission for Radiological Protection (ICRP)^1^ is based around the concept of ‘Reference Animals and Plants’ (RAPs) with the RAPs being defined at the taxonomic level of family. The ICRP proposes that the exposure of different life-stages should be considered in environmental assessments. One of the ICRPs RAPs is the Reference Frog (defined as the Ranidae family) for which, the ICRP propose, spawn (mass of eggs), tadpoles and adult life-stages be considered in assessments.

To estimate exposure there is a need to quantify activity concentrations in organisms including different life-stages and this is a recommendation of the ICRP^1^. However, in ICRP^2^, there were insufficient data to recommend transfer parameter values for life-stages other than adults; data for adult frogs (Ranidae species) were only presented for four elements (Ca, Cr, Pb and Zn) in the freshwater ecosystem^2^. Similarly, neither the International Atomic Energy Agency (IAEA) wildlife transfer parameter handbook^3^ or the revised version of the commonly used model for wildlife dose assessment, the ERICA Tool^4^, present transfer parameter values for Cs and amphibians in the freshwater environment. A number of post-Fukushima studies in Japan have investigated radiocaesium concentrations in frog species e.g.^5–8^. However, to our knowledge transfer parameter values for frogs in the freshwater environment have not been published for studies conducted in Japan.

Limited data (for on two adults and three tadpoles) presented in IAEA^9^ suggests that the transfer of radiocaesium to tadpoles is approximately one order of magnitude higher than that to adults. Data from Fukushima reported by Watanabe *et al*.^8^ supported the suggestion that radiocaesium activity concentrations in tadpoles would be higher than those in adult frogs. However, Watanabe *et al*.^8^ did not report water activity concentrations and transfer parameter values could not be derived.

**Figure 1:**
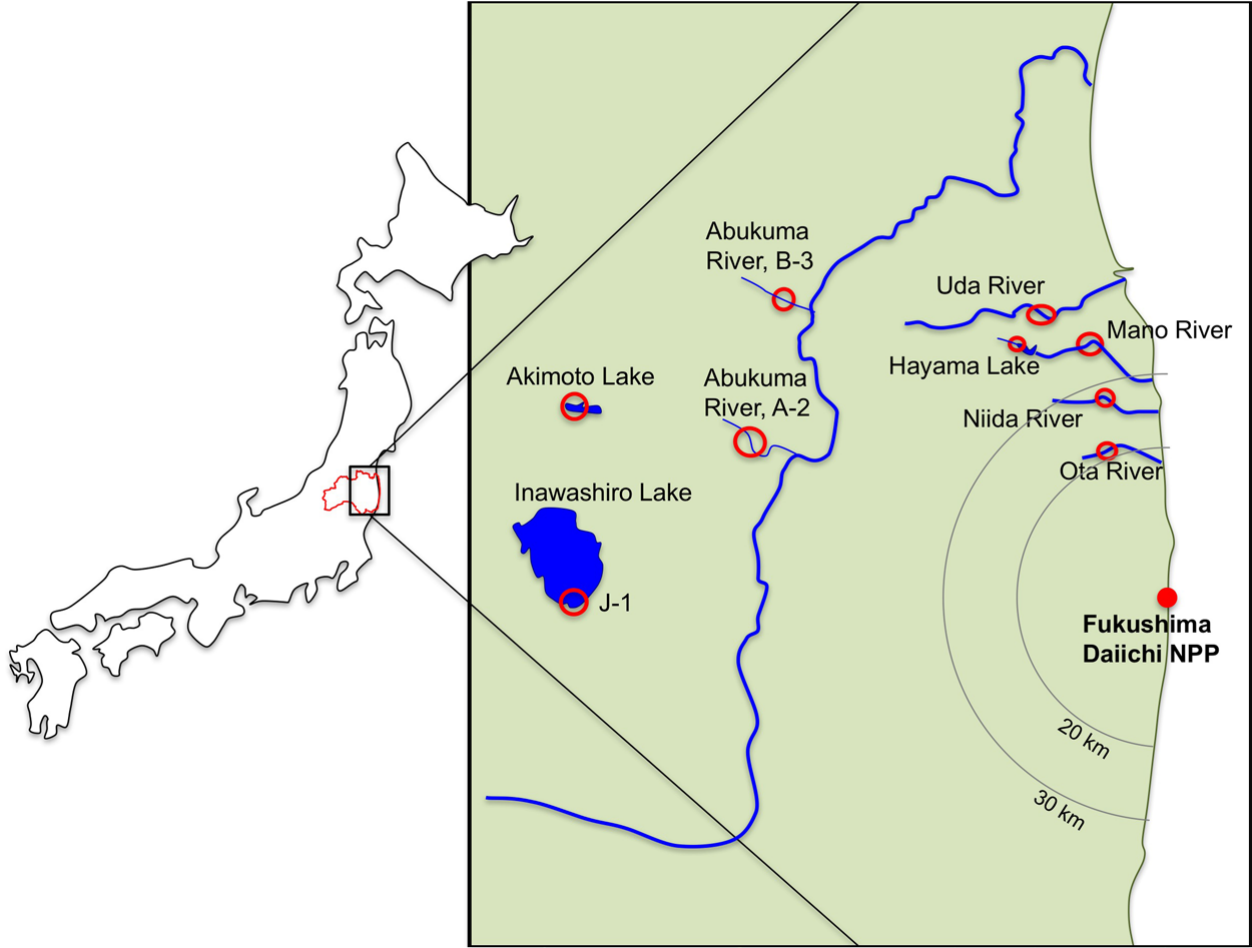
Frog and tadpole sampling sites in Fukushima (drawn from information presented by the Ministry of the Environment (http://www.env.go.jp/en/water/rmms/result_ao17-part.html)^10^ using Microsoft PowerPoint™ for Mac version 15.32).

In this study, we have used open source monitoring data for the Fukushima Prefecture^10^ to calculate transfer parameter values for radiocesium for both tadpoles and adult frogs (‘frog’ is used here to describe any member of the order Anura which includes true frogs and toads). Data were collated from five river systems (Abukuma River (two sites, A-2 and B-3), Mano River, Niida River, Ota River and Uda River) and three lakes (Hayama Lake, Aimoto Lake, and Inawashiro Lake) (Fig. 1). The transfer parameter values presented in this paper are concentration ratios (CR_wo-water_), which relate the whole-organism activity concentration (Bq kg^−1^ fresh mass) to the activity concentration in water (Bq l^−1^). We also use the data to evaluate the exposure of different life-stages for two frog species and put these into context with international recommendations on the effects of radiation on amphibians.

## Results

**Figure 2:**
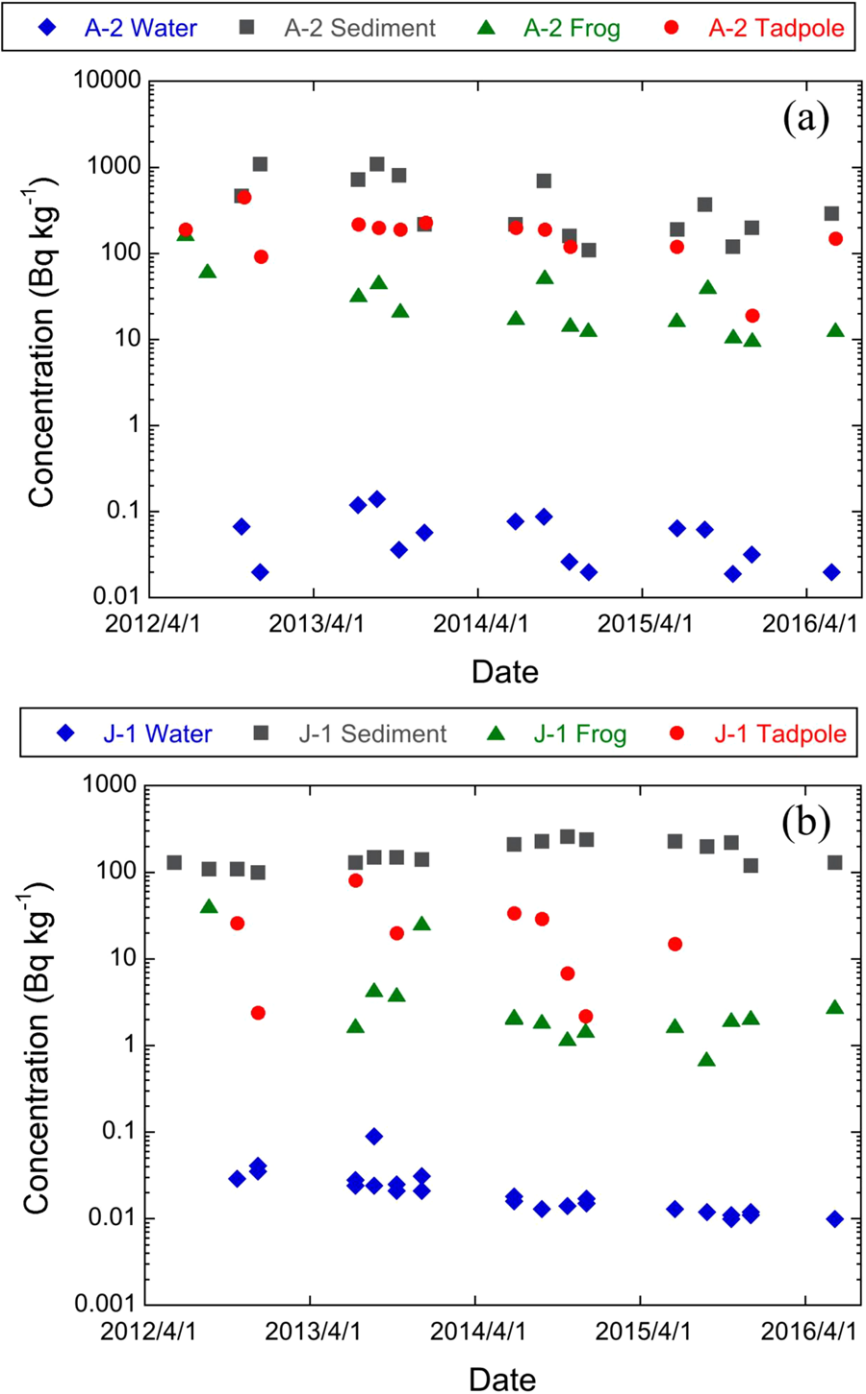
Activity concentration changes with time in ^137^Cs activity concentrations in water, sediment (dry matter), adult frogs and tadpoles (fresh mass) collected from (**a**) Abukuma River (site A-2) and (**b**) Inawashiro Lake (site J-1).

As an example of the collated data, Fig. 2 presents changes in ^137^Cs activity concentrations with time in water, sediments, adult frogs and tadpoles for Abukuma River and Inawashiro Lake (these were the two water bodies for which most data were available). The complete collated dataset is available as Supplementary Information. Data were available for: American bullfrog (*Rana catesbeiana*), Eastern-Japanese common toad (*Bufo japonicus formosus*), Forest Green frog (*Rhacophorus arboreus*), Japanese Brown frog (*Rana japonica*), Japanese tree frog (*Hyla japonica*), Kajika frog (*Buergeria buergeri*), Montane Brown frog (*Rana ornativentris*), Schlegel’s Green tree frog (*Rhacophorus schlegelii*), Tokyo Daruma pond frog (*Rana porosa porosa*), and Wrinkled frog (*Glandirana rugosa*)^10^ (Table 1). One measurement for each of adult frog and tadpole was reported as having a ^137^Cs activity concentration below detection limits (see Supplementary Information).

**Table 1:** Life-history information for American bullfrog, Montane Brown frog, Tokyo Daruma pond frog and Wrinkled frog in Japan.

Name	Adult frog size (mm)^29^	Breeding/life-span^29,41^	Habitat^29^	Adult diet^29^	Tadpole information^29,42,43^
American bullfrog (*Rana catesbeiana*)	Male: 111–178 Female: 120–183	May-September 6,000–40,000 eggs/season. Life-span 7–9 y	Plains and low hills, in association with paddy fields and larger water bodies with good vegetation cover at edges.	Carnivorous: Beetles, crayfish, tadpoles, small frogs, fish, mice, hatchling birds	Omnivorous eats dead leaves, algae, and plankton. Remain tadpoles over the winter
Montane Brown frog (*Rana ornativentris*)	Male: 42–60 Female: 36–78	January-June 1,000–1,900 eggs/season Life-span 3–4 y; takes 2–3 y to mature	Lowland plains and hillside, but most abundant at higher altitudes.	Carnivorous: Insects, snails, slugs, earthworms	Omnivorous eats waterweed and algae. Length 43–60 mm
Tokyo Daruma pond frog (*Rana porosa porosa*)	Male: 39–75 Female: 43–87	April-July 800–2,000 eggs/clutch Life-span 2–4 y; takes 2–3 y to mature	Lowland plains and rice paddies	Carnivorous: Insects, spiders, earthworms, slugs, land snails, frogs, small snakes	Likely omnivorous eating plant materials. Length 60 mm
Wrinkled frog (*Glandirana rugosa*)	Male: 37–46 Female: 44–53	May-August Up to 1,000 eggs/season Life-span 2–5 y; takes 1–2 y to mature	Favour marshes and rice paddies but also stream sides	Carnivorous: Spiders, insects (especially ants)	Omnivorous eats algae, etc. Mostly overwinter as tadpoles. Length 45–80 mm.

**Figure 3:**
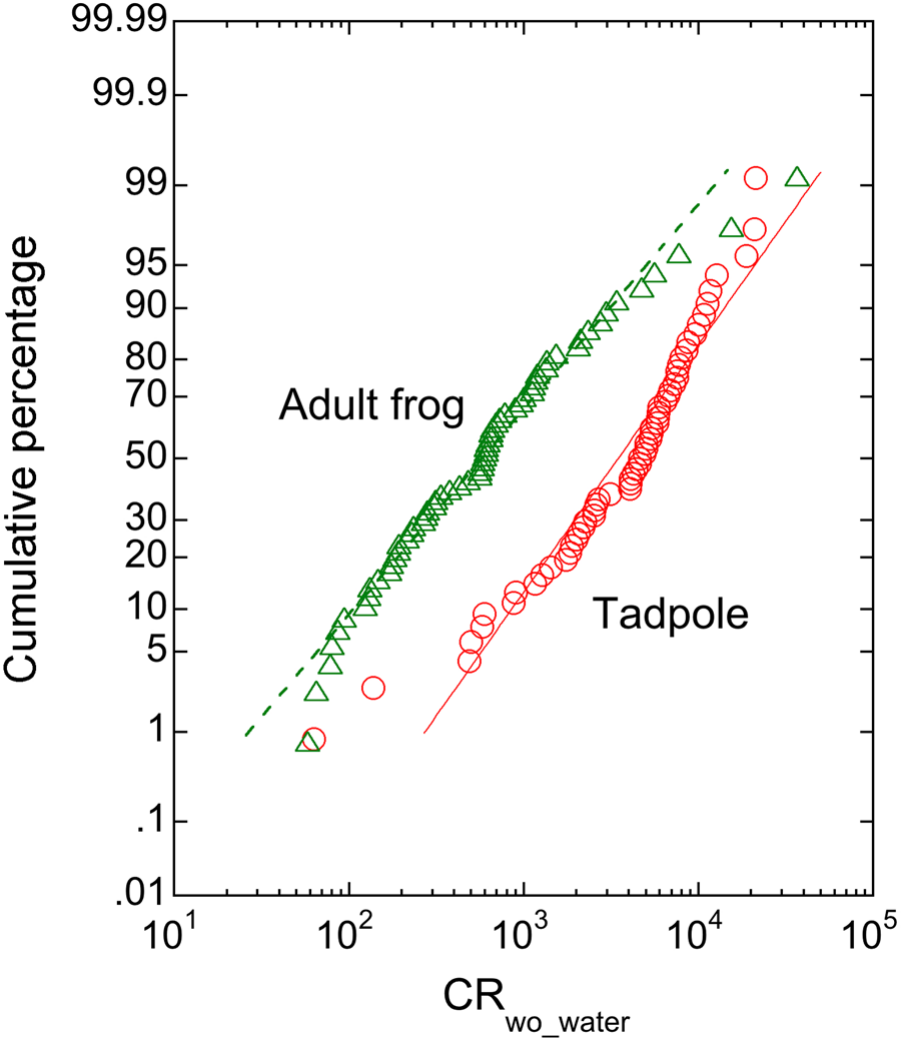
Distribution of CR_wo_water_ values (l kg^−1^ fresh mass) for ^137^Cs in tadpoles and adult frogs collected in the Fukushima freshwater environment 2012–2016.

Concentration ratios estimated from the collated data for frogs collected in the Fukushima Prefecture are presented, by species and life-stage, in Table 2. For adults, 62 CR_wo-water_ values were estimated, though individual CRwo-water values are based upon samples generally comprising of multiple individual frogs. For tadpoles, 59 CR_wo-water_ data were calculated, but tadpole species was generally not given. Where tadpole species was specified, it was possible to calculate tadpole CR_wo-water_ values for American bullfrog (n = 10), Kajika frog (n = 1) and Montane Brown frog (n = 1). Concentration ratio data generally have a lognormal distribution^11^. Graphical analysis of the tadpole and adult frog CR_wo-water_ data also tended towards log-normal (Fig. 3) and hence data were logged here prior to statistical analysis. Given there was only one CR_wo-water_ for each of adult frog and tadpole based on an organism activity concentration below detection limits, the ‘less than’ CR_wo-water_ values have been used in the subsequent analyses.

**Table 2:** Concentration ratio (CR_wo_water_; l kg^−1^ fresh mass) of ^137^Cs in adult frogs and tadpoles collected in Fukushima Prefecture, Japan, in 2012–2016.

Life stage	Species	n (samples)	n (individuals)	GM/AM	GSD	min	max
Tadpole	**All**	59	2540	3.5 × 10^3^	3.1	<6.3 × 10^1^	2.1 × 10^4^
	American bullfrog	10	77	5.9 × 10^3^	1.5	2.5 × 10^3^	1.2 × 10^4^
	Kajika frog	1	293	2.4 × 10^3^			
	Montane Brown frog	1	n/a	1.9 × 10^4^			
Adult	**All**	62	513	5.8 × 10^2^	3.8	5.8 × 10^1^	3.7 × 10^4^
	American bullfrog	6	8	5.7 × 10^2^	2.8	8.0 × 10^1^	1.5 × 10^3^
	Eastern-Japanese common toad	1	1	7.8 × 10^2^			
	Japanese Brown frog	2	24	3.3 × 10^2^		1.8 × 10^2^	4.8 × 10^2^
	Japanese tree frog	1	42	2.3 × 10^2^			
	Kajika frog	1	2	1.5 × 10^4^			
	Montane Brown frog	7	42	3.3 × 10^3^	3.9	5.9 × 10^2^	3.7 × 10^4^
	Tokyo Daruma pond frog	6	75	2.9 × 10^2^	2.0	1.2 × 10^2^	6.2 × 10^2^
	Wrinkled frog	24	211	3.5 × 10^2^	3.1	4.7 × 10^1^	5.6 × 10^3^

GM: geometric mean when n (samples) >2; AM: arithmetic mean when n (samples) = 2; single value quoted when n = 1; n/a = not available.

### CR_wo-water_ values for ^134^Cs vs ^137^Cs

For a given element, CR_wo-water_ values are generally assumed to be the same for all isotopes^2,3,12,13^, so CR_wo-water_ values calculated for ^137^Cs would be applicable to ^134^Cs. This hypothesis was tested here using all adult frog and tadpole data for which we had both ^134^Cs and ^137^Cs data reported above the detection limit. Paired T-tests confirmed no significant difference in the transfer of the two Cs isotopes for both the adult frogs (p = 0.12; n = 53) and the tadpoles (p = 0.48; n = 55). Given the isotopic independence of transfer, only ^137^Cs derived CR_wo-water_ values have been used in the subsequent data analysis presented here.

### Temporal variation in CR_wo-water_ values

**Figure 4:**
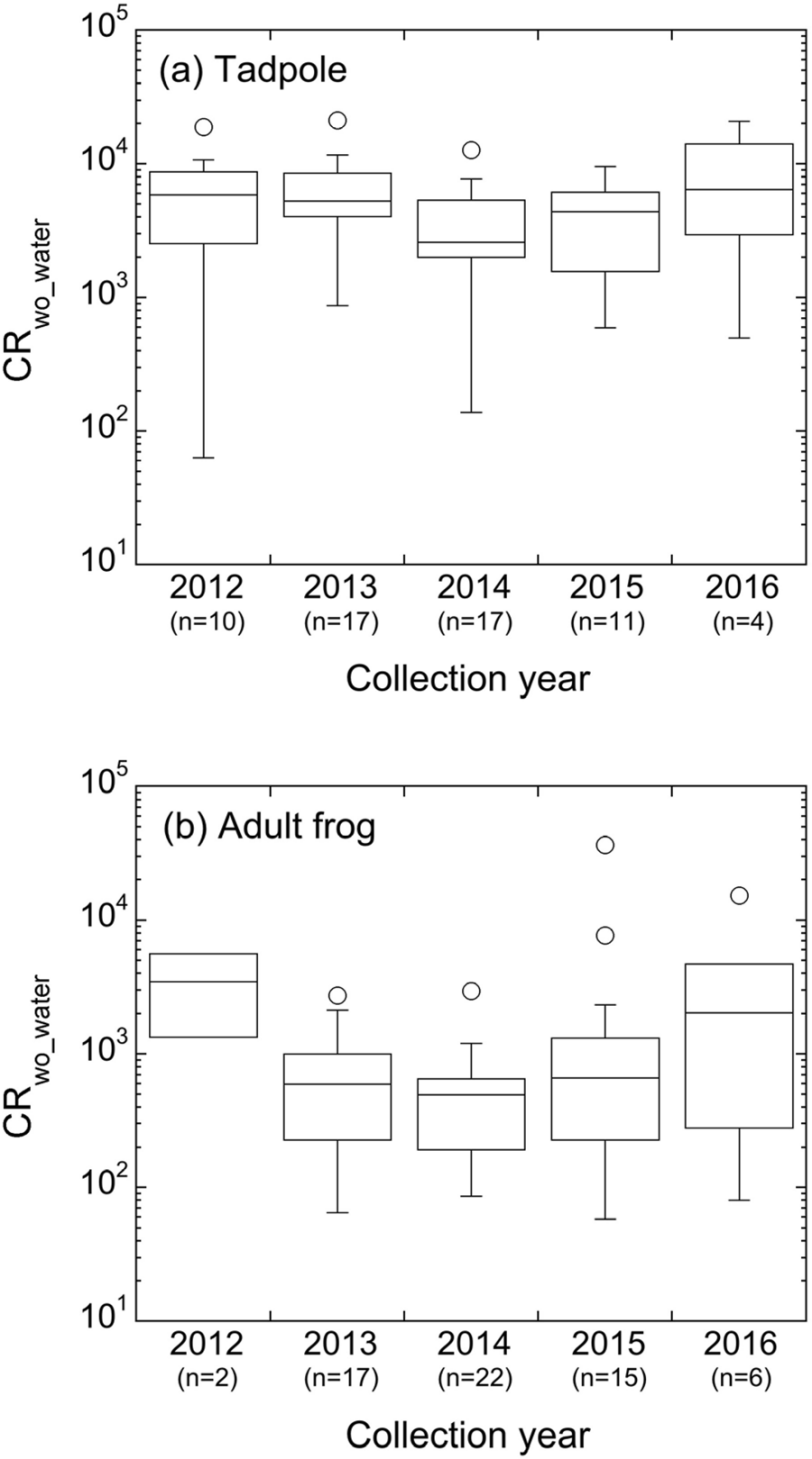
Box-whisker plots of annual C_wo_water_ values (l kg^−1^ fresh mass) for tadpole (**a**) and adult frog (**b**) collected in Fukushima Prefecture 2012–2016. Whiskers show −1.5 IQR of lower quartile and +1.5 IQR of upper quartile, and box shows lower and upper quartiles. Open circles represents outliers.

Figure 4 shows estimated tadpole and adult frog CR_wo-water_ values summarised by year. Only two adult frog CR_wo-water_ values could be calculated for 2012 as water data were lacking for other samples. For 2016, data were only available for the first month of annual sampling. A regression analysis across all individual sampling times over the period for which data were available revealed no statistically significant time trend in either the adult (r^2^ = 0.08) or tadpole (r^2^ = 0.01) CR_wo-water_ values. Therefore, it would appear that CR_wo-water_ had reached an equilibrium by 2012/13 so hereafter we analyse the data ignoring year of sampling.

The data were insufficient to allow analysis of any seasonal trend without the confounding influence of site.

### Comparison of CR_wo_water_ for tadpole and adult frogs

When data for all species were considered, the geometric mean (GM) value for tadpole (3.5 × 10^3^ l kg^−1^) was significantly higher than that for adult frog (5.8 × 10^2^ l kg^−1^) (T-test, p < 0.0001) (Table 2). Where sufficient data were available, this trend was also consistent across the different water bodies. For each of Abukuma River, Akimoto Lake and Inawashiro Lake, tadpoles (n = 24, 9, 9 respectively) had a significantly higher CR_wo-water_ value than adult frogs (n = 22, 11, 14 respectively) (T-test, p < 0.05).

**Figure 5:**
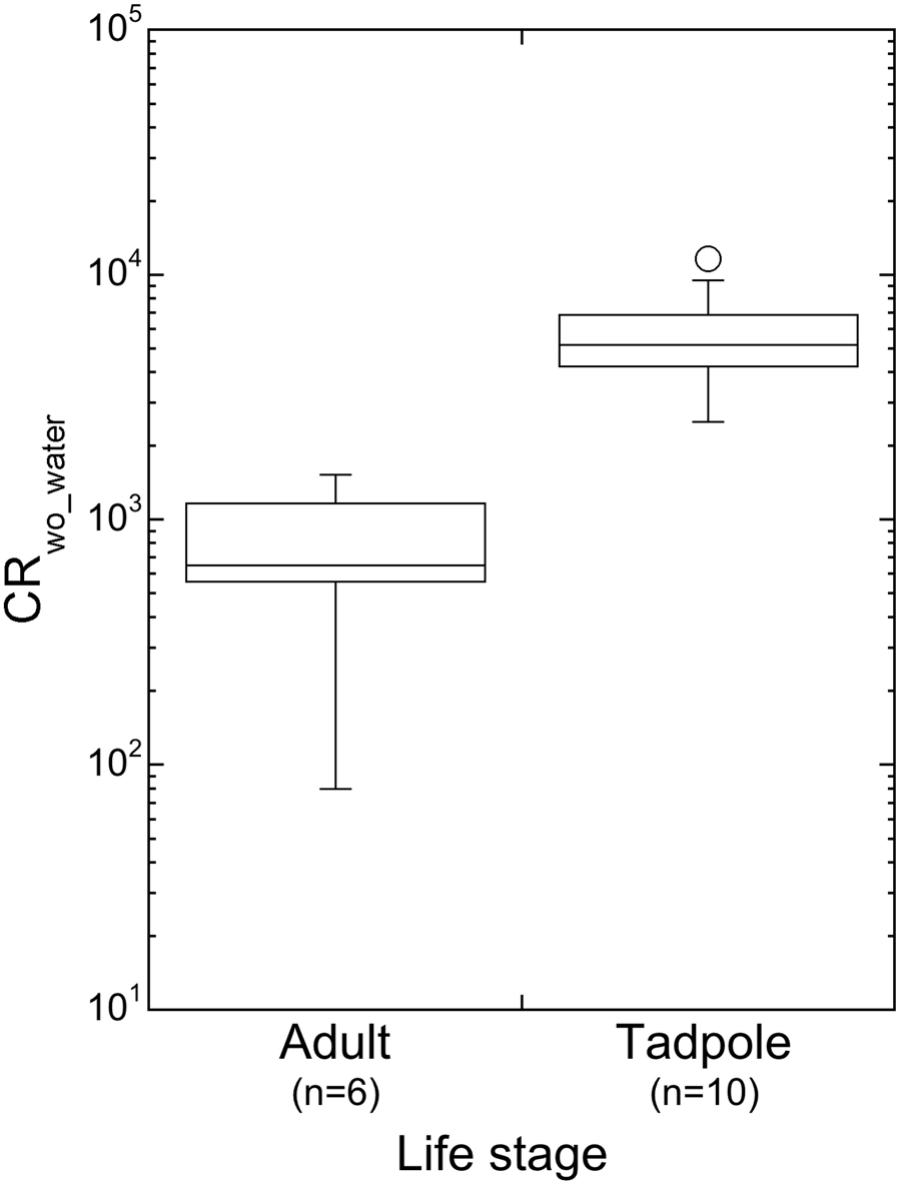
Comparison of CR_wo_water_ values (l kg^−1^ fresh mass) for American bullfrog adults and tadpoles collected in Fukushima Prefecture 2012–2016.

Although species was not identified for most tadpole samples, where this information was provided a similar trend was seen with tadpoles having a higher CR_wo-water_ than adults of the same species. For example, Fig. 5 presents data for American bullfrog for which the GM CR_wo-water_ value was approximately ten times higher for tadpoles than for adults (T-test, p = 0.002).

### CR_wo-water_ values – species variation

Adult frog CR_wo-water_ values for species with sufficiently high sample numbers (i.e. American bullfrog, Montane Brown frog, Tokyo Daruma pond frog and Wrinkled frogs) were compared using a General Linear Model. The Montane Brown frog was found to have a significantly higher CR_wo-water_ value than the other three species (p < 0.05).

### CR_wo-water_ values – lake vs river

Data for radiocaesium transfer to adult frogs were available for both lakes and rivers, presenting an opportunity to test whether there was a significant difference in the transfer between these two types of freshwater system. A T-test showed no significant difference between the CR_wo-water_ values for adult frogs in lakes (n = 26) and rivers (n = 36) (p = 0.19). However, for tadpoles the CR_wo-water_ value for rivers (n = 41) was significantly higher than that for lakes (n = 18) (p = 0.012; Mann-Whitney Test).

Figure 2, suggests there may be more seasonality in the activity concentrations of river water compared to lake water and this may hence add to additional variability within CR_wo-water_ values estimated for rivers. However, examination of the data (see Supplementary Information) shows no seasonal tend in CR_wo-water_ values for Abukuma River (the river for which there are most data).

### Estimated dose rates

Table 3 presents the estimated dose rates for the Montane Brown and Wrinkled frogs and their life-stages in Lake Akimoto in 2012 and 2016. Apart from estimates for the frogspawn life-stage, differences between the two species are minimal. As a consequence of the similarity in predictions for the two species at Lake Akimoto, dose rates for Uda River were only estimated for the Montane Brown frog (Table 4).

**Table 3:** A comparison of estimated absorbed dose rates to different life-stages of Montane Brown and Wrinkled frogs for Lake Akimoto in 2012 and 2016.

Year	Species	Dose µGy h^−1^					
		Frogspawn	Tadpole	Adult sediment-water interface	Adult in sediment	Adult on soil	Adult in soil
2012	Montane Brown	2.70E-03	3.47E-01	3.39E-01	6.54E-01	2.33E-01	5.71E-01
	Wrinkled	3.07E-04	3.47E-01	3.18E-01	6.33E-01	2.11E-01	n/a
2016	Montane Brown	7.39E-04	4.99E-01	4.86E-01	9.65E-01	1.18E-01	3.03E-01
	Wrinkled	8.29E-05	4.99E-01	4.80E-01	9.59E-01	1.12E-01	n/a

n/a – not applicable Wrinkled frog assumed not to hibernate in the terrestrial environment.

**Table 4:** A comparison of absorbed dose rates to different life-stages of Montane Brown frogs for Uda River in 2013 and 2015.

Year	Dose µGy h^−1^					
	Frogspawn	Tadpole	Adult sediment-water interface	Adult in sediment	Adult on soil	Adult in soil
2013	2.91E-03	1.17E-01	1.17E-01	2.05E-01	2.99E-01	7.45E-01
2015	1.11E-03	1.04E-01	1.06E-01	1.98E-01	1.57E-01	4.08E-01

## Discussion

The similarity in CR_wo-water_ values for ^134^Cs and ^137^Cs provides further evidence to support the commonly used approach^2,3,12,13^ of treating isotopes of a given element as having the same CR_wo-water_ value (if the isotopes can be assumed to be in equilibrium). The CR_wo-water_ values reported here show considerable variability when summarised as the generic frog or tadpole level. However, this variation is typical of that seen for CR_wo-water_ values of other organisms^3^. Whilst progress is being made in developing alternative approaches which account for the main cause of variability^14,15^ currently we are reliant of the CR approach and it is the parameter used in all environmental assessment models^16^ and proposed by international bodies^2,3^.

Although previous studies have suggested that tadpoles may have higher radiocaesium transfer than adults^8,9^, this suggestion was based on limited data. Our comparison of CR_wo-water_ values between adult frogs and tadpoles is, to our knowledge, the first conclusive demonstration that there is a significant difference in radiocaesium transfer for these two life-stages. Tadpoles are generally thought to be herbivorous or detritivorous^17^, whereas adult frogs are carnivorous (Table 1). Whilst we may expect radiocaesium to biomagnify up aquatic foodchains^18,19^ (in common with terrestrial foodchains^20,21^), however, tadpoles feed solely in the aquatic environment whereas frogs mainly feed in the terrestrial environment often consuming insects (Table 1). Furthermore, there is some evidence that some tadpoles, including those of the American bullfrog, are carnivorous^17^.

The higher CR_wo-water_ value for Montane Brown frog compared to other species may be due to differences in habitat utilisation; Montane Brown frogs frequent wet forest areas whereas the terrestrial habitats used by the other species are predominantly paddy fields or the area close to waterbodies (Table 1). Watanabe *et al*.^8^ previously reported that frog species living in forests had higher radiocaesium activity concentrations than those living in paddy fields. Kajika frogs live in similar areas as Montane Brown frogs^22^; the single sample of this species reported here also had a comparatively high CR_wo-water_ value (Table 2).

Whilst our data suggest potential differences in the transfer of radiocaesium to different frog species, we acknowledge that this is based on relatively few measurements (though a comparatively large number of individual frogs which were bulked to ensure that radiocesium activity concentrations could be determined). The potential differences in transfer to different frog species need to be further substantiated before species-specific values could be recommended for use in assessments. Currently, we suggest that generic amphibian and tadpole CR_wo-water_ values are used for screening assessments undertaken to establish that there will be no potential impact of ionising radiation under conservative assumptions^23^.

Our comparison of CR_wo-water_ values between lakes and rivers identified a significantly higher transfer of radiocaesium to tadpoles in rivers, but no difference in the transfer to adult frogs. The reasons for this are currently unclear, although we note that comparatively few tadpole observations were available for lakes. Whilst international data collations^13^ of wildlife CR_wo-water_ allow data to be categorised on the basis of lakes or flowing waters (i.e. river and streams) there has been little analyses of CR_wo-water_ values for the two categories (to our knowledge this has been limited to a consideration of fish^14^).

There is no information to suggest how environmental factors may influence the transfer of radiocaesium to frogs in aquatic ecosystems. However, the K status of a waterbody may be expected to impact on Cs transfer to amphibians, as has been demonstrated for fish^24,25^. Whilst we do not have K data for the water bodies from which amphibians were sampled, NIRS^26^ presents data (n = 442) for major rivers throughout Japan. The 5^th^ and 95^th^ percentiles of these data show K concentrations in water vary by an order of magnitude and could therefore, based upon data for fish, explain some of the variation in the dataset presented here.

The aim of the dose rate assessment was to consider the dose received by different life-stages and also consider the impact of life history (by considering two species) on the dose received; it was not intended as a definitive assessment of dose and risk to frogs in the study are. Dose rates for the Montane Brown frog and the Wrinkled frog (Table 3) were similar for the adult and tadpole life-stages. This is because external dose rate from soil or sediment is predicted to dominate the total dose (≥90%). The dose rate to frogspawn was estimated to be approximately one order of magnitude higher for Montane Brown frog than Wrinkled frog. External dose was also estimated to dominate the total ^137^Cs dose to *R*. *arvalis* in a Swedish wetland^27^. Frogspawn was assumed to be in the water column and to receive no exposure to contaminated sediment, hence differences in the CR_wo-water_ values assumed for the two species impact on the total dose rate. If frogspawn had been assumed to be on the sediment surface then the total dose rate would be similar to the other life-stages (i.e. two - three orders of magnitude higher); this demonstrates the importance of the occupancy assumption for this assessment. In 2012, predicted dose rates to the adult frogs in the terrestrial and aquatic ecosystems are similar for Lake Akimoto. However, in 2016 dose rates in the aquatic ecosystem are higher than those in the terrestrial ecosystem due to the increased sediment ^137^Cs activity concentrations in 2016 compared to 2012 (Table 5).

**Table 5:** Media ^137^Cs activity concentration data for water and sediment/soil (dry matter) as used for the dose assessments.

Waterbody	Year	Cs-134			Cs-137		
		Water (Bq l^−1^)	Sediment (Bq kg^−1^)	Soil (Bq kg^−1^)	Water (Bq l^−1^)	Sediment (Bq kg^−1^)	Soil (Bq kg^−1^)
Akimoto Lake	2012	0.019 [n = 1]	450 [n = 1]	424 [n = 1]	0.032 [n = 1]	760 [n = 1]	663 [n = 1]
	2016	0.003 [n = 1]	370 [n = 1]	119 [n = 1]	0.011 [n = 1]	2000 [n = 1]	663 [n = 1]
Uda River	2013	0.018 [n = 3, 0.0046]^+^	116 [n = 3, 117]	478 [n = 3, 14.5]	0.037 [n = 3, 0.0115]	249 [n = 3, 254]	1090 [n = 1]
	2015	0.005 [n = 2, 0.002–0.007]	86 [n = 2, 52–120]	242 [n = 2, 223–261]	0.016 [n = 2, 0.006–0.026]	355 [n = 2, 260–450]	1090 [n = 1]

^+^Range where n = 2, standard deviation where n = 3.

For Montane Brown frogs the period from egg to tadpole is reported to be *circa* 14 d and the tadpole life-stage to last about 120 d^28^; comparative values for the Wrinkled frog are cited as 5 d and 1 year respectively^29^. Therefore, from Table 3 it can be seen that the greatest contribution to lifetime dose is predicted to occur during the adult life stage; a similar conclusion was made for estimated 137Cs doses to R. arvalis in a Swedish wetland^27^. Furthermore, whilst the other life-stages may be more radiosensitive than adults^1^, their estimated absorbed dose rates are similar to, or considerably lower than, those for the adults.

Compared to Lake Akimoto, the predicted dose to adults at Uda River is higher in the terrestrial than aquatic environment (Tables 3 and 4). This is because the soil radiocaesium activity concentrations at this site are higher than those for sediment (Table 5), whereas at Lake Akimoto sediment activity concentrations are similar to, or higher than, those in soil.

Based on dose rates presented in Tables 3 and 4, if a four-month hibernation is assumed then this period (when it is assumed to be in sediments or underground) would result in a higher contribution to the annual dose of adult frogs than the remainder of the year.

For Lake Akimoto in 2012, ^137^Cs activity concentrations in water, sediment and soil were higher than ^134^Cs. However, the dose assessment demonstrated that for adults and tadpoles of both species ^134^Cs contributed a greater component of the total dose rate (*c*. 60%) than ^137^Cs in that year. This is due to the higher external dose conversion coefficient (relating Bq kg^−1^ in sediment to absorbed dose rate in µGy h^−1^) for ^134^Cs compared to ^137^Cs (Brown *et al*., 2016). For frogspawn, ^137^Cs dominates the total dose rate as a consequence of the low contribution of the external dose, higher activity concentrations of ^137^Cs compared to ^134^Cs in the environment and the higher internal dose conversion coefficient of ^137^Cs compared to ^134^Cs. Due to the shorter physical half-life of ^134^Cs (2.06 y) compared to ^137^Cs (30 y), at Uda River in 2013 the contributions of the two radiocaesium isotopes were similar and ^137^Cs contributed the most to doses for all life-stages in 2015/2016 at both sites (*c*. 70% of the estimated total dose rate).

Dose rates estimated for frogs at the two sites are considerably below the lower end of the Derived Consideration Reference Level (DCRL; ‘an order of magnitude dose rate band in which radiation induced effects might be expected to occur’) for amphibians of *c*. 40 µGy h^−1^ as suggested by ICRP^1^. Estimated dose rates are also at least an approximate order of magnitude below the generic 10 µGy h^−1^ no effects dose rate suggested by Andersson *et al*.^30^.

We acknowledge that there are some limitations to the dose assessment presented here. Whilst we used species-specific CR_wo-water_ values for the adult and frogspawn life-stages, a single value was used for tadpole for both species. However, the impact of this is likely to be minimal given the dominance of external exposure which contributed ≥90% of the total dose under the scenarios considered. Consequently, a change in the tadpole CR_wo-water_ by an order of magnitude (the approximate difference between the two species in CR_wo-water_ values for the adult life-stage; Table 2) is not going to effect the overall dose estimate greatly. For the assessment of frogs in the terrestrial ecosystem, we assumed the same organism activity concentration as predicted for the aquatic system given we lacked specific data to estimate CR_wo-soil_ values. Comparing the assumed wholebody activity concentrations to soil activity concentrations, predicted CR_wo-soil_ values in the range E-3 to E-1 can be estimated. Brown *et al*.^4^ report a CR_wo-soil_ value for amphibians of (4.6 ± 8.3)×10^−1^ (arithmetic mean value ± standard deviation; n = 139) from the latest version of the international Wildlife Transfer Database (WTD)^13^ which is a large compilation of CR_wo_ values initially established to help prepare IAEA and ICRP publications^2,3^. Whilst the values estimated here for Fukushima frogs look reasonable compared to this mean value they are at the low end of the observed data in the WTD (the range in WTD entries, which are a mixture of means and individual data entries, is E-2 to E + 0). However, CR_wo-soil_ values for a Ranidae species in Spain of E-3 to E-2 have recently been reported^31,32^. As noted in Materials and methods we used the ERICA Tool default amphibian geometry rather than creating specific geometries for the adult frog species considered as for the size range considered the actual geometry used has minimal influence on the dose rate estimated^33,34^.

In summary, the data presented here suggest that the transfer of radiocaesium to tadpoles is higher than that to adult frogs. This finding will be a useful input into the developing ICRP environmental protection framework as this considers various life-stages for the Reference Frog^1,2^, but lacks data to quantify transfer. Our CR_wo-water_ values represent some of the first reported; currently the wildlife models and data compilations do not contain CR_wo-water_ values for amphibians^2–4,13^. A CR_wo-water_ value of around 250, based on two observations, was reported in IAEA^9^. For seven out of the eight species considered here, the geometric mean CR_wo-water_ values were higher than this; the maximum values calculated here were about two orders of magnitude higher than that in IAEA^9^ (Table 2). Stark *et al*.^27^ reported CR_wo-water_ values for *Rana arvalis* in a Swedish wetland of 2500–19100 that are more similar to those we have calculated here. Estimated absorbed doses rates for tadpoles and the adult lifestage were dominated by external exposure. The adult life-stage was estimated to receive the greatest contribution of total lifetime dose. Estimated doses rates were below suggested effects benchmark dose rates for the two sites assessed. However, on the basis of data presented by Matsushima *et al*.^5^, dose rates to adult frogs in the most contaminated parts of Fukushima Prefecture may have exceeded the 10 µGy h^−1^ no-effect dose rate^30^ and at least approached the lower-limit of the ICRP’s DCRL^1^. Therefore, the Fukushima impacted areas present a natural laboratory for studying ionising radiation effects on frog populations (and indeed other wildlife).

## Materials and Method

We have extracted data from MOE (Ministry of the Environment)^10^ for the eight freshwater systems for samples collected between June 2012 and June 2016; the data included radiocaesium activity concentrations in water, sediment and biota. Corresponding soil data are also available from the terrestrial ecosystem adjacent to each site^10^; for rivers three to five soil cores were collected on each sampling occasion on either side of the river bank whilst for lakes five soil cores were collected around the sampling location^35^. The Ministry of the Environment use standard methods established by the Japanese Ministry of Education, Culture, Sports, Science and Technology (MEXT) (see MOE^36^). In brief, for sediments three replicate 5 cm cores were collected per site on each sampling occasion, these were dried and then homogenized and bulked into one sample prior to analyses. An approximately 20 l surface water sample was collected by bucket, if required this was filtered (1 µm) prior to concentration (by ammonium molybdophosphate coprecipitation^37^ or evaporation) for subsequent analyses. Biota samples were washed with de-ionised water, the gastrointestinal tract removed and homogenised for subsequent analyses. Due to the low mass of individual tadpoles and of many adult frogs, samples collected were often bulked for a given sampling site prior to analysis (see Table 2 and Supplementary Information). Eleven samples of adult frogs were a composite of two to five species. Three samples had no species information recorded. A total of 513 adult frogs and 2540 tadpoles were analysed in 62 and 59 composite samples respectively.

Gamma-emitting radionuclides were determined in all sample types using Ge-detectors.

### Calculation CR_wo-water_ values

The transfer parameter values presented in this paper are concentration ratios (CR_wo-water_), which relate the whole-organism activity concentration (Bq kg^−1^ fresh mass) to the activity concentration in water (Bq l^−1^). To calculate CR_wo-water_ values, water data collected at the same sampling site as frog samples were used. Water data from the collection date closest to the biota sampling date were used to calculate CR_wo-water_ values; if the biota sampling was between the water sampling dates, then the average water activity concentration was calculated. In all cases, the difference in sampling date between water and biota samples used to calculate a CR_wo-water_ value was less than 1 month.

Caesium-137 and ^134^Cs activity concentration data were available for both adult frogs and tadpoles. However, ^134^Cs was undetectable in many of the water samples so only the ^137^Cs data have been used for calculating radiocaesium transfer parameter values. For ^137^Cs one measurement was under the detection limit for both frog and tadpole (both samples from Inawashiro Lake); the detection limit value was used for the calculation of CR_wo-water_.

### Estimation of dose rates

Dose rates have been estimated for the Wrinkled frog and Montane Brown frog given they have somewhat different life-histories and are examples of a species with a comparatively low and high CR_wo-water_ values respectively. Assessments have been conducted for Akimoto Lake and Uda River for both the first and last years that water activity concentration data were available. The CR_wo-water_ for each species (Table 2) was used to estimate wholebody ^134^Cs and ^137^Cs activity concentrations; the overall tadpole CR_wo-water_ value was used for both species given the lack of relevant species-specific data. No data were available for the transfer of Cs to frogspawn (eggs) for the sites considered here. Therefore, to allow a dose estimate for all life-stages we have assumed the CR_wo-water_ for frogspawn was 0.09 times the CR_wo-water_ for the adult of each species based upon data from the Chernobyl Exclusion Zone^38^ and a forest site in northeast England (Beresford & Barnett CEH pers comm.). Given the lack of any specific data, the wholebody radiocaesium activity concentrations for adult frogs were taken to be the same in the terrestrial environment as that predicted in the aquatic environment.

The ERICA Tool^4^ was used to estimate absorbed dose rates. When not hibernating it was assumed that the adult frog was on the soil surface in the terrestrial environment and at the sediment-water interface in the freshwater environment; tadpoles were modelled at the sediment-water interface and frogspawn in the water column. It was assumed that the Montane Brown frog hibernates in either soil or (aquatic) sediment whereas the Wrinkled frog hibernates in sediment only^39,40^. For adults of both species the default amphibian geometry from the ERICA Tool was used (this is the same as the Reference Frog geometry proposed in ICRP^1^); it has been demonstrated^33,34^ that there is minimal influence of size on dose within the size ranges of the frogs sampled here and the ERCIA default and hence there is no need to create organism in the Tool for each species. For frogspawn and tadpoles, new geometries were created in the ERICA Tool. The geometry presented for frogspawn mass in ICRP^1^ was used for this life-stage, and the tadpole geometry was based on information for the two species being assessed (assumed mass = 3 g, length = 6 cm, width and height = 1 cm).

### Statistical analysis

All statistical analyses were performed using MINITAB version 17; the tests used are quoted in the text. MINITAB does not quote levels of significance below 0.0001, hence any p-values below this are quoted as p < 0.0001. By necessity statistical analyses had to be based upon the number of composite samples analysed rather than the number of individual animals analysed.

### Data statement

All individual data (activity concentrations in different sample types, as extracted from MOE (http://www.env.go.jp/en/water/rmms/result_ao17-part.html)^10^, and estimated CR_wo-water_ values) are available as supplementary information.

## Electronic supplementary material

Supplementary Information
